# Identifying Targets for Interventions to Increase Earplug Use in Noisy Recreational Settings: A Qualitative Interview Study

**DOI:** 10.3390/ijerph182412879

**Published:** 2021-12-07

**Authors:** Michael T. Loughran, Samuel Couth, Christopher J. Plack, Christopher J. Armitage

**Affiliations:** 1 Manchester Centre for Health Psychology, School of Health Sciences, University of Manchester, Manchester Academic Health Science Centre, Manchester M13 9PL, UK; chris.armitage@manchester.ac.uk; 2 Manchester Centre for Audiology and Deafness, School of Health Sciences, University of Manchester, Manchester Academic Health Science Centre, Manchester M13 9PL, UK; samuel.couth@manchester.ac.uk (S.C.); chris.plack@manchester.ac.uk (C.J.P.); 3 Department of Psychology, Lancaster University, Lancaster LA1 4YF, UK; 4 Manchester University NHS Foundation Trust, Manchester Academic Health Science Centre, Manchester M13 9QN, UK; 5 NIHR Greater Manchester Patient Safety Translational Research Centre, University of Manchester, Manchester M13 9PL, UK

**Keywords:** hearing protection interventions, hearing conservation, hearing protection behaviour, behaviour change, recreational noise-induced hearing loss, COM-B model, TDF, qualitative research

## Abstract

Earplugs can reduce the risk of hearing loss and tinnitus. However, earplug use during noisy recreational activities is uncommon, and methods for increasing uptake and regular use have had limited efficacy. The aim of the present study was to examine barriers and enablers of ever-performers (e.g., people who have used earplugs) and never-performers (e.g., people who have not used earplugs) to identify targets to inform the content of interventions to increase uptake and regular use of earplugs in recreational settings. The Capabilities, Opportunities, and Motivations model of Behaviour (COM-B) informed the outline for 20 semi-structured telephone interviews (ever-performers, *N* = 8, age range = 20–45 years; never-performers, *N* = 12; age range = 20–50 years). Thematic analysis was used to identify barriers and enablers to earplug use, which were mapped onto the Theoretical Domains Framework (TDF). Six key domains of the TDF were identified. Ever-performers described being more exposed to ‘*social influences*’ (e.g., facilitators such as friends/peers) and were more positive than never-performers concerning ‘*beliefs about consequences*’ (e.g., earplug protection outweighs any negative effects on listening/communication). Involvement of ‘*emotion*’ (e.g., fear of losing ability to listen to music) and ‘*reinforcement*’ tactics (e.g., creating habits/routines) were discussed by ever-performers, but were not mentioned by never-performers. Both groups reported lack of ‘*environmental context and resources*’ (e.g., prompts and cues), and their own ‘*memory, attention, and decision processes*’ (e.g., deciding when to use earplugs) as barriers to earplug use. The present research identifies the variables that would need to change in order to increase earplug uptake and use in recreational settings among ever-performers and never-performers. Further work is required to translate these findings into testable interventions by selecting appropriate intervention functions (e.g., *modelling*), policy categories (e.g., *communication/marketing*), behaviour change techniques (e.g., *demonstration of behaviour*), and mode of delivery (e.g., *face-to-face*).

## 1. Introduction

Noise-induced hearing loss (NIHL) and tinnitus due to recreational noise exposure has become a major public health concern [1,2,3]. Recent epidemiological studies have shown that both younger and older adults are exposing themselves to potentially hazardous recreational noise across multiple settings [4,5]. These risks could be mitigated through use of earplugs [6,7], but a recent population survey estimated that only 2% of UK adults regularly used hearing protection during noisy recreational activities [4]. Further, a recent systematic review found few robust intervention studies to improve hearing protection behaviours and concluded that evidence-based interventions were needed [8]. The aim of the present research was to examine barriers and enablers to earplug use in order to inform interventions to improve uptake and regular use of earplugs in recreational contexts. 

In the context of developing interventions to promote earplug use in recreational settings, Hunter [9,10] found barriers to earplug use to include social pressure and lack of aesthetic appeal; as well as the belief that wearing earplugs would worsen the listening experience, impair communication, and be uncomfortable. However, sampling was limited to young adults [9,10] and those who had diagnosed hearing problems [10], yet it has been shown that older adults also regularly participate in noisy activities [4,5,11], and it would be more valuable to develop interventions prior to hearing problems. Beach et al. [12] focused on the opinions of those who almost always used earplugs in nightclubs, yet there are multiple recreational contexts in which earplugs could be used that contribute to overall exposure [5]. 

In addition to the limitations outlined above, previous research in this area has typically not been underpinned by relevant theories of behaviour change. When the overarching aim is to gain insights to develop interventions that improve a desired health related behaviour, the use of theory facilitates the summarising of evidence, helps identify what needs to change [13], and can lead to better intervention outcomes [14]. The exception was Beach et al. [12], who mapped findings onto the constructs of the Health Belief Model (HBM; see [15,16]), concluding that personal experience of hearing symptoms (e.g., hearing loss and tinnitus) was the most common enabler, or trigger, of earplug uptake. Mapping also revealed enablers to include an awareness of the benefits of earplugs, long-term negative effects of hearing loss on quality of life, and confidence in abilities to wear earplugs correctly [12]. However, attending nightclubs is just one recreational context, and little is known about the drivers of behaviour for people who do not use earplugs. Accordingly, it would be valuable to directly compare the barriers and enablers of those that have previously used earplugs (termed ‘ever-performers’ from herein); (e.g., [8,17]), across a range of recreational activities, compared to those that have never used earplugs recreationally (termed ‘never-performers’ from herein). Ultimately, these insights will help to inform intervention development to improve uptake and sustained use [8]. 

The Capabilities, Opportunities, and Motivation model of Behaviour change (COM-B) [18,19] is designed to capture all the major drivers of behaviour, including those not captured in theories such as the HBM. COM-B comprises six components, namely: (1) psychological capability, (2) physical capability, (3) physical opportunity, (4) social opportunity, (5) automatic motivation, and (6) reflective motivation. Underpinning COM-B is the Theoretical Domains Framework (TDF) [18,20] that more precisely elucidates 14 potential determinants of behaviour (e.g., knowledge, goals, social influences, intentions) synthesised from 33 behaviour change theories. By undertaking interviews constructed around COM-B and analysing them using the TDF [21], it is possible to identify what needs to change with respect to earplug use in recreational contexts, similar to that reported for musicians in occupational contexts [22]. To date, no study has conducted interviews with ever-performers and never-performers of earplugs in recreational contexts to gain insights into behaviour utilising the COM-B model and TDF. 

Thus, the aim of the present study was to examine and compare barriers and enablers of earplug use for noisy recreational activities for ever- and never-performers, in order to identify potential determinants of behaviour that can inform interventions to increase uptake and use of earplugs in recreational contexts.

## 2. Materials and Methods

### 2.1. Study Design and Recruitment 

This study used semi-structured telephone interviews to gather the data. Recruitment was aimed at anyone aged 18–69 years who took part in noisy recreational activities (e.g., found themselves in noisy situations other than the workplace), and was conducted via social media advertising, university promotion through email, websites, and posters. People who opted for interview were contacted via email with a participant information sheet explaining the process. A £10 gift voucher was offered to anyone completing an interview. Ethics approval was granted by the Division of Psychology and Mental Health, University of Manchester, June 2018. Reference: 2018-3556-6133. Interviews took place between June 2018 and June 2019. 

### 2.2. Participants

Participants were 20 adults aged between 20–50 years, of whom eight had previously used earplugs (either ‘always’, ‘often’, ‘sometimes’, ‘seldom’; see [23]) within any recreational context (e.g., ‘going to nightclubs or pubs’, ‘outdoor sports’; see [24]), for any length of time (i.e., ‘ever-performers’) and 12 who had never used earplugs recreationally (i.e., ‘never-performers’). No ever-performers used earplugs in all noisy recreational situations. Participant demographics are shown in Table 1. 

### 2.3. Interview Process and Data Collection

Verbal consent and anonymity were maintained throughout. Telephone interviews were performed within the lead researcher’s office, and lasted approximately 30 min. Initially participants were asked about their loudest recreational experiences derived from the amount of vocal exertion required in order to communicate with others [23,25], followed by being asked if they used earplugs within these scenarios. Participants were then asked about barriers and enablers of earplug use, structured around the COM-B model, and developed by authors MTL and CJA aided by guidance from the appropriate literature [18] (Supplementary Material S1). 

Exploration of people’s psychological capability included questions about knowledge of earplugs; education; and knowing when to use them. Questions about the physical skill of using earplugs; knowing how to use them-and if not, where to get relevant information-were used to explore physical capability. Physical opportunity focused on the physical environment and resources people have been exposed to, such as: do they know where to get earplugs; physical prompts and cues regarding earplugs; and affordability of earplugs. The social influences afforded to people define the way they think about things, and questions on these influences as well as social stigma and conformity assessed social opportunity. Automatic motivation questions were difficult to contextualise appropriately, and we therefore based these questions on the ‘self-report behavioural automaticity index’ subscale [26]; meaning responses based on impulse/habit could encourage closed responses (yes/no). Reflective motivation assessed people’s intentions and evaluations to engage with earplugs after a period of reflection. Full outline questions can be seen in Supplementary Material S1; however, flexibility was left to the discretion of the lead researcher during the interview process.

### 2.4. Data Analysis

Transcripts were organised and coded using Nvivo 11 and 12 software (QSR International, Melbourne, Australia). Participants were separated into ‘ever-performers’ or ‘never-performers’ of earplugs. 

Independently, interview data were analysed thematically by the first author (MTL), with secondary analysis then carried out by the second author (SC); involving the six phases outlined by Braun and Clarke [27]. All initial coding, collapsing of relationships between codes and themes, and mapping to the COM-B and TDF took place independently, and was finalised through meetings. Due to comparisons between ever- and never-performers, themes were applied to one or both groups to describe the data fully, highlighting the barriers and enablers of earplug use in noisy recreational contexts (see Supplementary Material S2 and Table 2 and Table 3). The fourth author (CJA) reviewed these codes and themes throughout and helped resolve any disagreements, while the third author (CJP) reviewed the final decisions. Authors MTL and SC independently mapped thematic analysis themes to the COM-B. As questions were developed using the COM-B, initial codes reflected these constructs, but as codes were collapsed into barrier and enabler themes it became apparent that certain themes spanned more than one COM-B component, as seen previously in Boyd et al. [28]. Mapping was determined through likelihood of ‘fit’ with the COM-B based upon the description and definitions from Michie et al. [18]. Similarly, themes within COM-B components were mapped to the relevant ‘fit’ of the 14 TDF domains, based upon the descriptions and definitions outlined by Michie et al. [18]. This process allows the targeting of precise behavioural determinants for the implementation of evidence-based interventions [18]. COM-B and TDF mapping was agreed between authors MTL and SC, with CJA and CJP crosschecking and resolving any disagreement.

## 3. Results

Within this section, we have presented a blended interpretation of the data based around the domains of the TDF to highlight potential determinants to target for future interventions. Barrier and enabler data broadly fitted within 13 domains of the TDF. The authors agreed on six key dominant domains (‘*social influences*’, ‘*environmental context and resources*’, ‘*beliefs about consequences*’, ‘*memory, attention, and decision processes*’, ‘*reinforcement*’, and ‘*emotion*’) across five COM-B components (Supplementary Material S3). Verdicts on rank of importance were decided upon between the authors based on, (1) which domains suggest clear differences between the two groups in driving behaviour, and (2) which domains need to be targeted for both groups in future interventions aimed at improving uptake and sustained use. The remaining seven TDFs (‘*knowledge*’, ‘*intentions*’, ‘*goals*’, ‘*optimism*’, ‘*physical skills*’, ‘*social role and identity*’, and ‘*belief about capabilities*’) are mentioned as consequence of the six key domains. Full descriptions of barriers and enablers can be seen in Supplementary Material S3, with corresponding links to the COM-B and TDF found in Table 2 and Table 3.

### 3.1. Key TDF Domains

#### 3.1.1. Social Influence (Social Opportunity)

Ever-performers described face-to-face/group advice and encouragement from credible sources (e.g., friends, family, peers) as a driver of earplug use. Communication about earplugs (e.g., benefit, style, impact on listening) or personal/shared experiences of hearing symptoms by social influences (e.g., social opportunity), no matter how brief, has helped to initiate some form of behaviour change. Never-performers show no evidence of social support to normalise behaviour; however, they appear open to these influences in the future.

“*My younger brother, he was a drummer. So he’d come along to the gig with me and my partner, and had gone, oh yeah I’ve got these earplugs that I use when I’m drumming and I just take them to all gigs now, and it’s really great because you can still hear the band but when you come out of it you’re not completely deaf. And I was like, oh that actually seems alright, you know, that’s probably the thing to do.*” **P#9 (ever)**

“*I’ve never even thought about it and I don’t know what it’d be like. I’ve never had any friends say, hi, you should try this as well, but sometimes peers conversations is very helpful to gauge what things are like.*” **P#8 (never)**

Both groups reported other influences such as, social stigma still playing a role in people not performing the behaviour, especially group norms (e.g., wearing earplugs on a night out; never-performers), and alienation/judgement (ever-performers). Negative social norms link to lack of motivation (e.g., ‘*beliefs about consequences*’, ‘*social role and identity’ and ‘reinforcement’*) to perform behaviour; however, both groups indicated seeing others use earplugs has the potential to alter motivation.

“*I’ve been told off for it a couple of times*.” **P#10 (ever)**

“*I think some people probably think you might look weird if you do it, especially in the night club.*” **P#19 (never)**

“*Yeah, maybe I would start using them if friends and family started.*” **P#20 (never)**

#### 3.1.2. Environmental Context and Resources (Physical Opportunity) 

Generally, exposure to environmental contexts and resources (e.g., physical opportunities) was similar for both groups. Either group did not report physical prompts and cues (e.g., signs within environments, on tickets) to encourage/assist/promote/reinforce earplugs through person/environment interaction. However, while no one was able to recall seeing any prompts or cues, this does not mean they were not present.

“*No, there’s never any. There’s always about strobe lights, they always have a health warning, but there’s never a health warning about hearing, not that I’ve seen.*” **P#2 (ever)**

“*If they were offered at the entrance, I would probably think, okay, maybe that’s particularly loud and maybe I should wear these*.” **P#7 (never)**

“*Just it’s warning me of the dangers, so I would be more inclined to do something.*” **P#20 (never)**

Availability of material resources (e.g., earplugs) within recreational contexts was restricted to staff, not advertised, or limited to single use foam earplugs, which can be viewed negatively with respect to listening quality (see ‘*beliefs about consequences*’; see [29]). According to both groups, availability would encourage use, indicating ‘*intentions*’ and ‘*goals*’ (e.g., reflective motivation) to use earplugs in the future. Such willingness links to participants own ‘*beliefs about capabilities*’ (e.g., reflective motivation) about performing the behaviour, and suggests they are ‘*optimistic*’ (e.g., reflective motivation) about the benefits.

“*They all seem to have them behind the bar for bar staff, so yeah. I tend to go and ask. Even now, if I’ve forgotten the specific ones that I’ve got that I take to a gig, then I will go and get the foam ones as they…well, it’s better than nothing.*” **P#18 (ever)**

Consistent with Hunter [9] never-performers had the opinion that earplugs would lack comfort; however, comparatively ever-performers found earplugs to be comfortable (see ‘*beliefs about consequences*’). Both groups believe they have the necessary skills (‘*physical skills*’; physical capability) to use earplugs correctly, although it was not possible to objectively measure ever-performers’ and never-performers’ skill levels in the current study. Correct placement is associated with comfort and performance [30].

“*When they’re actually in my ear they felt fine. Comfortable enough. I mean, I’ve got musician earplugs so I’d like to hope they were designed to be comfortable*.” **P#1 (ever)**

Compared to never-performers, ever-performers appear to have had better access to resources (e.g., never-performers suggested chemists/pharmacies as a point of contact to buy earplugs, which may not stock the best quality/high-fidelity earplugs), and altered their own environments to assist performance of behaviour (e.g., placement of prompts and cues; also see ‘*reinforcement*’ and ‘*memory, attention, and decision processes’*).

“*I’d probably go to the chemist or the supermarket and hunt around the shelves.*” **P#12 (never)**

“*In terms of actually taking them from my house to a game or a practice, then I have a bum bag or fanny pack. If I remember to take them before I go out to a gig then I will get them out of the bum bag and then hang that on the door, so when I get back in again the bag is there and really obviously reminding me to put them back in again afterwards*.” **P#18 (ever)**

“*Yeah, I have a special little pouch now that I put my earplugs in, it’s like a pretty little pouch so they’re not just shoved in my bag so then I remember to take them with me*.” **P#21 (ever)**

#### 3.1.3. Beliefs about Consequences (Reflective Motivation)

Although ‘*knowledge*’ of hearing loss due to noise exposure exists within both groups (see ‘*memory, attention, and decision processes*’), personal hearing health appears to be a low priority (e.g., hearing taken for granted). Particularly never-performers, who undertake no pre-planned preventative actions, and indicate no current ‘*intentions*’ or ‘*goals*’ to protect.

“*Probably, but not in the near future, maybe in 10–15 years’ time when, I don’t know, I’ll be thinking, okay, I’m going to grow old soon, I might as well try to preserve my hearing.*” **P#7 (never)**

Hypothetically speaking, never-performers have similar negative *beliefs about consequences* to those discussed by Hunter [9], that earplugs will have a detrimental impact on listening, diminishing the overall listening experience, affect communication, and be uncomfortable (see ‘*environmental context and resources*’).

“*Yeah, like, the issue is that it’s too loud, and putting plugs in or headphones on will make the whole thing quieter, but I might as well just not be listening.*” **P#6 (never)**

In contrast, ever-performers have beliefs that are more positive. They find both foam and high-fidelity earplugs to be comfortable; however, those that used foam earplugs mentioned ’*knowledge*’ of high-fidelity earplugs, and that they may perform better, and may be more comfortable [29]. Ever-performers believe that earplugs are capable of reducing the risks of noise exposure (e.g., ‘*beliefs about capabilities’* and ‘*optimism’*), giving confidence of enjoying music for longer (see ‘*emotion*’ and ‘*reinforcement*’). While they do describe that wearing earplugs does alter how they hear and listen to pleasurable sounds, they believe this is not at the expense of continued enjoyment of activities. Their beliefs and actions indicate that they have ‘*intentions’*, ‘*goals*’, ‘*optimism*’, and a ‘*belief about capabilities*’ to perform protective behaviour. In some cases this belief can be applied to personal and shared experiences of hearing symptoms (see ‘*social influences*’).

“*When I’ve worn them, I can, definitely…you can hear the benefit because you…like I mean, they seem pretty decent, you can still hear everything it doesn’t…you don’t lose any of the music. You can still hear and talk to people*.” **P#5 (ever)**

“*I can enjoy what I’m trying to hear better without being overwhelmed by a wall of sound.*” **P#13 (ever)**

#### 3.1.4. Memory, Attention, and Decision Processes (Psychological Capability) 

Certain aspects of decision making and memory recall (e.g., psychological capability) present a barrier for both groups, such as: knowing that certain activities are loud enough to damage hearing, ‘how loud is too loud’ to warrant the use of earplugs, forgetting earplugs (ever-performers), and not taking action post hearing symptoms (e.g., temporary tinnitus; never-performers) (e.g., ‘*beliefs about consequences’*, ‘*environmental context and resources’* and ‘*knowledge*’). Both groups made decisions based on their subjective assessment of the circumstances (e.g., type of activity or environment, noise levels, length of exposure, frequency of attendance), and when faced with two alternatives (‘protect’, or ‘do not protect’) never-performers’ memory, attention, and decision processes led them to not using earplugs, as they have never given it that much attention.

“*I know I should and I know it’s something that I’ve not really thought about.*” **P#8 (never)**

Ever-performers are prone to making decisions that indicate poor judgement (e.g., not wearing in all noisy situations); however, it is also clear that they have the capacity to make appropriate decisions based upon the fact that they use earplugs ‘some of the time’, indicate forward planning (also see ‘*reinforcement*’, ‘*belief about consequences*’, ‘*intentions*’, and ‘*goals*’ (e.g., reflective motivation)), understand the benefits of earplugs (see ‘*beliefs about consequences*’), and recall memory of personal or shared experiences (see ‘*social influences*’).

“*It depends on the gig, there are some gigs where going in I know I’m going to want them throughout, so I’ll put them in. Others I may, sort of you know… say there are three of four bands playing, I may know which ones I’ll need it for and which ones I don’t, I’ll make that decision when they start.*” **P#13 (ever)**

“*No, I think I didn’t take any action because with the nightclubs I still carried on. I didn’t need earplugs. I think with the motorway, when I was driving on a motorway that was a routine habit already so it didn’t change any of my actions in anyway.*” **P#3 (ever)**

Participants’ ‘*knowledge*’ indicates psychological capability in understanding hearing can be protected with earplugs during noisy activities, without any evidence of formal hearing health education (e.g., ‘*knowledge*’).

“*My understanding of it is that if you do it [use earplugs] consistently, especially when you’re young, so around my age, it will sort of delay hearing loss when you’re growing older. It’s obviously not the only factor, but it’s one of the factors that will sort of make it less likely for you to become impaired or in terms of hearing when you become older, if you take measures early to protect your hearing, that’s my perception of it.*” **P#1 (ever)**

“*I like to keep healthy, but I guess I haven’t really seen my ears as part of my health as such in a sense, like I eat healthy and stuff like that, fruit and veg, but I haven’t really considered…there’s not much about–well, there’s not much promotion about the dangers of noise.*” **P#20 (never)**

#### 3.1.5. Reinforcement and Emotion (Automatic Motivation) 

Emotional responses were noted by ever-performers, with rationale including love of music (e.g., fear of hearing loss affecting enjoyment; also see ‘*belief about consequences*’), and general anxieties of acquiring symptoms. Love of music creates an incentive to protect hearing (e.g., longevity of enjoyment of music). This drive to protect involves anticipated regret, leading to automatic responses not evident in never-performers, and consequentially relates to ‘*intentions*’ and ‘*goals*’ (reflective motivation) to use earplugs (also see ‘*beliefs about consequences*’). With the exception of aesthetics (e.g., appearance of earplugs), positive automatic responses (‘*emotion*’ and ‘*reinforcement’*) to use earplugs were not reported by never-performers.

“*I know that it’s very important, ‘cause you don’t want to lose your hearing, especially if you like music as much as me.*” **P#2 (ever)**

The use of prompts and cues (see ‘*environmental context and resources*’) by ever-performers further increases the probability of performing the behaviour automatically (‘*reinforcement*’), through the creation of habits/routines/impulses, and building further reflective motivation through planning (e.g., ‘*intentions*’), and setting ‘*goals*’ (also see ‘*beliefs about consequences*’). Judgement and punishment for using earplugs was described by both groups as a barrier to use (see ‘*social influences*’), reinforcing negative automatic impulses not to wear earplugs. One noteworthy tactic used by an ever-performer to overcome this was to place earplugs in-situ before arriving at noisy recreational environments, removing the opportunity of judgement, social stigma (e.g., observation by strangers putting earplugs in, and concealment of earplugs by wearing their hair down), and increasing overall probability of use.

“*If people see you putting your earplugs in and sometimes people will be a bit like, oh what are you doing, you know. Judgemental. So definitely when I was younger, I’d put them in before I got to somewhere, and then wear my hair down so it would hide them.*” **P#21 (ever)**

As well as removing elements of social stigma, and perhaps unbeknownst to the participant, this act also facilitates adjustment to changes in their perceptions of sounds (e.g., habituation) before participation in an enjoyable activity, meaning that when they are exposed to the sound of their chosen activity, the transition may be easier and viewed as less detrimental (e.g., removing negative motivations; see *beliefs about consequences*). 

### 3.2. Summary of Findings

There are several prominent findings from these results to consider framed within the constructs of the TDF. Ever-performers’ interactions with *social influences* (e.g., credible sources) have been a major driving force helping to initiate earplug use when compared to never-performers. Both groups discussed *environmental context and resource* concerns within the UK, suggesting that there is poor availability of earplugs for patrons in venues, and no noticeable warnings about noise levels and the need to use hearing protection. Ever- and never-performers’ *beliefs about consequences* about of using earplugs differ; ever-performers believe that any changes (e.g., reduction of sound) incurred from using earplugs are secondary to the perceived benefits (e.g., longevity of hearing); while never-performers believe that such changes are barriers to use. The *memory, attention, and decision processes* of both groups have highlighted concerns regarding judgement of noise levels, and assessing when it is loud enough to use earplugs. Longevity of hearing and ability to enjoy music have evoked *emotion* in ever-performers to protect against excessive noise exposure, something not noted in never-performers, while this desire has encouraged *reinforcement* through routines (e.g., use of prompts) and impulses that are increasing ever-performers’ probability of using earplugs.

## 4. Discussion

This is the first qualitative study to examine barriers and enablers of earplugs to identify potential determinants of behaviour of interventions for use during noisy recreational activities by comparing the opinions and experiences of ever- and never-performers. Interview questions were informed by the COM-B model [18] and mapping of themes was underpinned by the TDF [20,31]. Wide-ranging opinions and experiences were condensed into six key determinants of behaviour from the TDF (*social influences; environmental context and resources; beliefs about consequences; memory, attention, and decision processes; reinforcement; emotion*) that may provide a platform for future interventions targeted at increasing uptake and sustained use of earplugs in noisy recreational situations. 

Our findings have identified the influence of social facilitators/credible sources (e.g., friends/peers) directly changing earplug behaviour of ever-performers through face-to-face discussions about hearing health, and modelling of earplugs. Interventions may benefit from the implementation of *social influences,* due to the behavioural impact described by ever-performers. To the best of our knowledge, this is the first time high levels of social involvement have been clearly identified as driving the enablement of recreational earplug use. Widén [32] suggested that social norms may impact on use of hearing protection, while Hunter [9] suggested that peer norms impact on people not wearing earplugs (e.g., no one else wears them). However, neither study focused on the individual value of credible sources enabling behaviour; Widén [32] stated that their social norm conclusion may lack validity; while Hunter [9] did not incorporate health psychology theory, and had limited views from ever-performers. Hence, Hunter [9] lacked social enabler content. 

In order for the behaviour to occur there must also be a physical opportunity (*environmental context and resources;* [18]), and our findings have revealed two clear targets for both ever- and never-performers that could be addressed in future interventions: (1) A lack of clear prompts/cues to use earplugs, and (2) poor availability of earplugs. UK guidelines state [33] that if an event is expected to be loud (>96 dBA) attendees should be made aware via messages (e.g., tickets, signs on entry) with the view to protect their hearing. With continuous noise levels regularly surpassing this requirement [34,35,36,37,38] such warnings should be clearly observable. Availability of earplugs was absent or limited to staff, potentially due to earplugs not being mandatory for attendees of recreational events in the UK [33]. Both groups suggested that better availability would encourage behaviour, similar to that recorded at Canadian rock concerts [39] and music events in the Netherlands [40]. However, availability is no guarantee of use [41], and earplug quality may be an important factor to overcome negative ‘*beliefs about consequences,’* with cheaper foam earplugs deemed least favourite when compared to high-fidelity earplugs due to comfort and appearance [29]. Nonetheless, distribution of both foam and high-fidelity earplugs within interventions has yielded better uptake compared to interventions that have not [8]. 

Comparison of ever- and never-performers’ *beliefs about consequences* revealed a more positive outlook from ever-performers. Personal hearing health was a low priority in the short-term for never-performers, who even after presence of hearing symptoms did not consider using earplugs. Similar to previous research, experience of hearing symptoms enabled ever-performers [12], but concerns over comfort and listening quality remain barriers for never-performers [9]. Preconceptions such as these are not unfounded, as earplugs do attenuate sound levels by varying amounts [42,43], can create localisation problems [44], occlusion effects [45], affect speech intelligibility [46], and are linked with comfort concerns [47]. However, although ever-performers admit that sound was attenuated, their positive beliefs about using hearing protection (e.g., comfort, activities remained pleasurable, hearing was protected/risk of hearing symptoms minimised) outweigh the negatives, and has led to better intentions and goals. *Beliefs about consequences* map directly with reflective motivation of the COM-B model [18], and these findings can be applied within the context of a recent survey [17] that suggests that never-performers lack reflective motivation compared to ever-performers, helping to further diagnose what needs to change in terms of behavioural determinants. 

*Memory, attention, and decision processes* is the ability to retain information, and when in an environment use that information to make behavioural decisions. Although both groups were not able to recall any formal education, they indicated adequate levels of knowledge on the dangers of noise causing hearing symptoms, and the benefits of earplugs mitigating the risks of exposure. However, only ever-performers were capable of making appropriate decisions to use earplugs, albeit not in every noisy recreational situation, and at times forgetting their earplugs. Both groups discussed that they felt certain situations were not loud enough to wear earplugs, even though the vocal exertion required when having a conversation suggests otherwise [23,25,48]. Unfortunately, subjective decision making on loudness is difficult, as each individual context will be different to the next [34,35,49], and it is not something that can be easily taught. However, there are other means to improve people’s *memory, attention, and decision processes*, for example, the delivery of information or warnings previously discussed within lack of *environmental context and resources*. 

Ever-performers described the *emotion* associated with fear of losing hearing, and not being able to enjoy pleasurable activities, such as music, enabling behaviour (e.g., anticipated regret). Beach et al. [12] described participants’ love for music, and although this theme did not fit within the constructs of the HBM, theoretical implementation of the COM-B and TDF has associated this now reoccurring theme with *emotion* (TDF) and an automatic motivation (COM-B model; [18]) to use earplugs. Automatic motivation also involves *reinforcement* (TDF) increasing the probability of using earplugs, but there was no evidence of this discussed by never-performers. Development of habits and desire to continue to enjoy music has created incentives and impulses (*reinforcement*) for ever-performers to use earplugs. These habits, impulses, and routines are key to creating automatic motivations to perform a behaviour, and can only be achieved if people have the desire to use earplugs. The recent survey by Loughran et al. [17] suggested that ever-performers have greater automatic motivation to use hearing protection compared to never-performers, and due to the mapping capabilities of the COM-B and TDF we can now associate this finding with the involvement of both *emotion* and *reinforcement*.

In order to address these barriers, enablers, and proposed TDF determinants, it would be beneficial to apply these theoretical findings alongside other notable research [8,17] to a systematic framework, such as the Behaviour Change Wheel (BCW; see [18]), which allows designers of interventions to move from a behavioural analysis to an intervention design, similar to that applied to the promotion of hearing aids [50]. If we use ‘*social influences*’ as a worked example, according to the BCW [18], the next steps towards a systematic design based on evidence would be selecting appropriate intervention functions (intervention categories; e.g., *enablement; modelling*), policy categories (policies to deliver chosen functions; e.g., *communication/marketing; service provision*), behaviour change techniques (smallest active ingredient of an intervention; e.g., *social support; demonstration of behaviour*), and a mode of delivery (e.g., *face-to-face*). It must be noted that this worked example is very simplistic, and a complex intervention will contain multiple interacting components potentially derived from various sources of evidence [51].

### 4.1. Strengths

The findings from this study provide a robust theoretical analysis base for future interventions to increase use of earplugs in noisy recreational settings. We discovered six key targets for behaviour change interventions to improve earplug use: *Social influences, Environmental context and resources, Beliefs about consequences, Memory, attention, and decision processes, Emotion, and Reinforcement*. Unlike previous qualitative studies, we have compared and contrasted both ever-performers’ and never-performers’ opinions and experiences, allowing us to specify differences in determinants. Incorporation of a theoretical model (COM-B) and a theoretical framework (TDF) adds strength to this study. An informed interview schedule utilising the COM-B model of behaviour has helped to capture all possible components driving behaviour, as opposed to a schedule informed by TDF, as rigid use could result in missing determinants [52]. However, the TDF has allowed more robust mapping of themes to behavioural determinants that can be integrated in developing interventions.

### 4.2. Limitations

Like most qualitative studies, our sample size was small (*n* = 20) and so cannot be viewed as a true representation of UK adults. However, the views expressed within a qualitative dataset can be used to compliment large-scale quantitative data [17], and help to inform the design of interventions that are representative of target populations. 

Investigating earplug use in multiple recreational contexts could be argued as a disadvantage due to various types of noise and sound pressure levels [34,35,49]; however, there were very few inconsistencies in people’s responses to engage with earplugs across multiple contexts, therefore underpinning that barriers and enablers are comparable across the sample. Our initial analysis recovered 13 determinants of the TDF involved in enacting earplug use, which we condensed to six key determinants to present in our results; however, it is important to acknowledge that future work may reveal that some of the seven determinants deemed less prominent in our analysis may also provide a pivotal role in changing behaviour.

## 5. Conclusions

For the first time, use of the TDF within the context of earplug use in noisy recreational contexts has provided a robust theoretical evidence base for the development of future interventions. Interventions that target ‘*social influences*’, ‘*environmental context and resources*’, ‘*beliefs about consequences*’, ‘*memory, attention, and decision processes*’, ‘*reinforcement* and *‘emotion*’ are a priority for increasing uptake and regular use of earplugs recreationally. Future research should prioritise the systematic development of testable interventions incorporating and expanding on theory-based evidence through frameworks such as the BCW.

## Figures and Tables

**Table 1:** Participant characteristics.

Characteristics	Ever-Performers *n* (%)	Never-Performers *n* (%)
Gender		
Female	5 (62.5)	7 (58.3)
Male	3 (37.5)	5 (41.7)
Ethnicity		
White	8 (100)	9 (75)
Black, Asian, Minority Ethnic	0 (0)	3 (25)
Age range		
20–29	3 (37.5)	7 (58.3)
30–39	3 (37.5)	3 (25)
40–49	2 (25)	1 (8.3)
50–59	0 (0)	1 (8.3)

**Table 2:** Earplug barriers and COM-B/TDF links.

Barrier Theme	Group	COM-B Links	TDF Links
***Circumstantial (e.g., noise type or environment, volume, length of exposure, frequency)***	Both	Physical opportunity	*Environmental context and resources*
		Psychological capability	*Knowledge* *Memory, attention and decision processes*
***Detrimental impact on listening***	Both	Physical opportunity	*Environmental context and resources*
		Reflective motivation	*Beliefs about consequences*
***Forgetting***	Ever	Physical opportunity	*Environmental context and resources*
		Psychological capability	*Memory, attention and decision processes*
***Incorrect use***	Never	Physical capability	*Physical skills*
***Lack of awareness and education***	Both	Physical opportunity	*Environmental context and resources*
		Psychological capability	*Knowledge* *Memory, attention and decision processes*
		Reflective motivation	*Beliefs about consequences*
***Lack of comfort***	Never	Physical opportunity	*Environmental context and resources*
		Psychological Capability	*Knowledge*
		Reflective motivation	*Beliefs about consequences*
***Lack of shared experiences***	Never	Psychological capability	*Memory, attention and decision processes*
		Social opportunity	*Social influences*
***Lack of prompts and cues***	Both	Automatic motivation	*Reinforcement*
		Physical opportunity	*Environmental context and resources*
		Social opportunity	*Social influences*
***Low priority***	Both	Automatic motivation	*Reinforcement*
		Psychological capability	*Memory, attention and decision processes*
		Reflective motivation	*Beliefs about consequences* *Goals* *Intentions*
***Not automatic to use earplugs***	Never	Automatic motivation	*Emotion* *Reinforcement*
***No preventative planning***	Never	Reflective motivation	*Beliefs about consequences* *Goals* *Intentions*
***Poor accessibility***	Never	Physical opportunity	*Environmental context and resources*
		Psychological capability	*Knowledge*
***Social stigma, norms, conformity etc***	Both	Automatic motivation	*Reinforcement*
		Reflective motivation	*Beliefs about consequences* *Social role and identity*
		Social opportunity	*Social influences*

**Table 3:** Earplug enablers and COM-B/TDF links.

Enabler Theme	Group	COM-B Links	TDF Links
***Ability to use earplugs***	Ever	Physical capability	*Physical skills*
***Advice and encouragement (social facilitators/credible sources)***	Both	Social opportunity	*Social influences*
		Reflective motivation	*Social role and identify*
***Aesthetics not important***	Never	Automatic motivation	*Emotion* *Reinforcement*
***Affordable***	Ever	Physical opportunity	*Environmental context and resources*
***Availability***	Both	Physical opportunity	*Environmental context and resources*
		Reflective motivation	*Beliefs about capabilities* *Goals* *Intentions* *Optimism*
***Awareness of NIHL and hearing protection behaviours***	Both	Psychological capability	*Knowledge* *Memory, attention and decision processes*
***Circumstantial (e.g., noise type or environment, volume, length of exposure, frequency)***	Both	Physical opportunity	*Environmental context and resources*
		Psychological capability	*Knowledge* *Memory, attention and decision processes*
		Reflective motivation	*Goals* *Intentions*
***Comfort***	Ever	Physical opportunity	*Environmental context and resources*
		Psychological capability	*Knowledge*
		Reflective motivation	*Belief about consequences*
***Earplug performance***	Ever	Reflective motivation	*Beliefs about consequences* *Beliefs about capabilities* *Optimism*
***Earplug routine and planning***	Ever	Automatic motivation	*Reinforcement*
		Physical opportunity	*Environmental context and resources*
		Psychological capability	*Memory, attention and decision processes*
		Reflective motivation	*Beliefs about consequences* *Beliefs about capabilities* *Goals* *Intentions*
***Experiences with hearing symptoms***	Ever	Psychological capability	*Knowledge* *Memory, attention and decision processes*
		Reflective motivation	*Beliefs about consequences* *Goals* *Intentions* *Optimism*
		Social opportunity	*Social influences*
***Good quality earplugs***	Ever	Physical opportunity	*Environmental context and resources*
***Knowledge of earplugs***	Both	Physical opportunity	*Environmental context and resources*
		Psychological capability	*Knowledge*
		Reflective motivation	*Beliefs about consequences*
***Love of music***	Ever	Automatic motivation	*Emotion* *Reinforcement*
		Reflective motivation	*Beliefs about consequences* *Goals* *Intentions*
***Perceived benefits of using earplugs***	Ever	Psychological capability	*Knowledge* *Memory, attention and decision processes*
		Reflective motivation	*Beliefs about consequences* *Goals* *Optimism*
***Prompts and cues***	Ever	Automatic motivation	*Reinforcement*
		Physical opportunity	*Environmental context and resources*
		Psychological capability	*Memory, attention and decision processes*
		Social opportunity	*Social influences*

## Data Availability

The datasets used and/or analysed during the current study are available from the corresponding author on reasonable request.

## Supplementary Materials

The following are available online at https://www.mdpi.com/article/10.3390/ijerph182412879/s1, S1—Qualitative Interview Structure; S2—Barriers and Enablers; S3—TDF and COM descriptions.
