# Supplementary material for: Identifying Targets for Interventions to Increase Earplug Use in Noisy Recreational Settings: A Qualitative Interview Study
Source: Int J Environ Res Public Health. 2021 Dec 7;18(24):12879. doi: 10.3390/ijerph182412879 (PMC8701360; doi:10.3390/ijerph182412879)
Supplement: Supplementary file 1 [file ijerph-18-12879-s001.zip › SF1 - Qualitative Interview Structure.pdf]

SF1 – Qualitative Interview Structure

Interview Introduction

Hello, my name is [interviewer] and today we are going to chat about wearing earplugs during noisy activities that you might take part in outside of work. A "noisy activity" is one in which, if you were 4 feet (1.2 metres) away from someone with normal hearing, you would have to raise your voice to speak to them, but noisy activities do not include use of headphones/earphones. Noisy activities can cover a wide range of settings such as: live music (e.g., concerts, festivals), nightlife (e.g., clubs, bars, pubs), making music (e.g., in a band, home producer), DIY (e.g., power tools, powered gardening tools), engine noise (e.g., motorbikes, motorboats, motorsports), sports related noise (e.g., watching rugby or football matches live, firearms and fireworks) or cinema. However, during the interview you are not restricted only to these examples.

[Confirm and discuss hearing protection use: always, often, sometimes, seldom, never]

<u>**Experiences questions**</u>

Always

- Activities: ________ ________ ________ ________ ________
- Why did you always wear earplugs during [**activity**]?
- Did you ever want to stop wearing them during **[this activity**]? If so, why?
- How long have you been wearing earplugs for **[this activity**]?
- Do you have any routines/habits that you follow before or after you wear earplugs for **[this activity**]?
- How did it make you feel after using earplugs for **[this activity**]?
- Do you think earplugs give you peace of mind for **[this activity**]?
- Do you ever believe earplugs to be unnecessary for **[this activity**]?

- Do you think earplugs enhanced your experience, diminished or made no difference for **[this activity**]? If so, why?
- **[Discuss this question after having gone through all the activities above and if not already been asked]**
- If you were trying to persuade someone to start wearing earplugs for noisy activities, how would you go about doing it?

Often/Sometimes/Seldom

- Activities: ________ ________ ________ ________ ________
- Why did you not wear earplugs all the time during [**activity**]?
- Did you ever start and stop wearing them during **[this activity**]? If so, why?
- How long have you been wearing earplugs while doing **[this activity**]?
- Do you have any routines/habits that you follow before or after you wear earplugs for **[this activity**]?
- How did it make you feel after using earplugs for **[this activity**]?
- Do you think earplugs give you peace of mind for **[this activity**]?
- Do you ever believe earplugs to be unnecessary for **[this activity**]?
- Do you think earplugs enhanced your experience, diminished or made no difference for **[this activity**]? If so, why?

[Discuss these two questions after having gone through all the activities above]

- If you were trying to persuade someone to start wearing earplugs, how would you go about doing it?
- What kinds of things do you think would persuade you always to wear earplugs for noisy activities?

Never

- Activities: ________ ________ ________ ________ ________
- Why did you never wear earplugs during **[this activity]**?
- Did you ever think about wearing earplugs during **[this activity]**? If yes, why? If no, why?
- Would you have worn earplugs if they had of been available or if you had brought your own during **[this activity]**?
- Do you believe earplugs to be unnecessary for **[this activity]**?
- Do you think earplugs would enhance your experience, diminish or make no difference for **[this activity]**? If so, why?

[Discuss this question after having gone through all the activities above and if not already been asked]

- What kinds of things do you think would persuade you to start wearing earplugs for noisy activities? How would you try and persuade others to wear them?

[ALL participants asked the following question, referring to activities]

Now trying to think about the noisy activities, during these do you ever think to yourself "this is too loud"?

If yes - Which activities did you find too loud?

[Prompt for more than one example if deemed necessary]

Do you have any particular examples of times that stand out?

Which one particular activity stands out as being the loudest?

Did you do anything about it being too loud?

If no - On reflection now what situation do you think was the loudest?

__COM-B Questions__

[Ask secondary questions when appropriate]

Psychological Capability

Generally, what do you know about protecting your hearing during noisy activities?

Do you know why it is sometimes a good idea to wear earplugs during noisy activities?

To elaborate on the last point, are you aware of the dangers of noise during noisy activities?

If yes - What do you know about the dangers?

Where or how did you learn about this information?

If no - Do you think there is any particular reason why you don't know about any dangers of noise exposure?

Have you ever been given any advice on protecting your hearing with devices such as earplugs?

If yes - Where did you get this advice?

Did you understand the advice?

Did the advice then make you take any action?

If no - Why do you think this may be the case?

Have you ever thought to yourself before or during noisy activities that perhaps it may be loud; I might need to do something to help protect my ears?

If yes - Why did you think this?

What did you then do about the situation?

If no - Why do you think this is the case?

Do you know approximately how long it takes for hearing damage to occur due to noise exposure before it is noticeable?

Who do you think is responsible for protecting your hearing during recreational activities (e.g., self-responsibility/government body)?

Physical Capability

Do you know how to place earplugs correctly in your ears?

If yes - How did you know how to do it?

Did you find it easy?

Do you think placement is important?

If no - Would you know where to get such advice/information?

Do you think it would be easy?

Do you think placement would be important?

Do you know what it feels like to wear any form of earplug in your ears?

If yes - What were your thoughts on how it felt?

Any positive or negative feelings as to how it felt?

If no - Do you have any thoughts on what it might feel like?

Do you think there could be any positive or negatives in terms of how it may feel?

Physical Opportunity

Do you know where you can get/buy earplugs?

If yes - Could you please elaborate? **[If not already]**

Do you know or have you seen if there are different types of earplugs?

If yes - Can you describe these to me?

Do you know how much earplugs can cost?

If yes - Can you tell me how much?

Do you think earplugs are an affordable form of hearing protection?

If yes - Why is this?

If no - Why is this?

Have you ever had access to earplugs during activities?

If yes - What were these situations?

Did this make a difference for you?

If no - Do you think these should be available?

Would this make a difference for you?

Have you ever been aware of any cues/signs indicating that an activity may be noisy? This can be either before or during that would prompt you to use earplugs.

If yes - What were these?

Did this make a difference for you?

If no - Do you think these should be available?

Would this make a difference for you?

Social Opportunity

Have you ever had any views on how earplugs look?

If yes - What are your opinions?

Would these views ever make you reconsider using or not using them?

If no - Why is this?

Do you think how they look would make you reconsider whether or not to use them?

Within your social circle do you know anyone who wears earplugs regularly?

If yes - Has this ever given you any thoughts about wearing them?

Overall within your social circle how many friends would you say wear earplugs?

If no - Why do you think this is the case?

Do you think seeing people within your social circle wearing earplugs would in any way influence you?

Within your social circle did you all ever have a conversation about how an activity was loud and that perhaps we should wear earplugs?

If yes - Can you tell me more about this?

Did this ever make a difference for you or any of the group?

If no – Why do you think this is?

Would this make a difference if you all did?

Automatic Motivation

Is wearing earplugs something you do automatically?

If yes - Why is this?

If no - Why do you think this is the case?

Is wearing earplugs something you do without thinking?

If yes - Why is this?

If no - Why do you think this is the case?

Is wearing earplugs something you do without consciously remembering?

If yes - Why is this?

If no - Why do you think this is the case?

Is wearing earplugs something you start to do before you realise you are doing it?

If yes - Why is this?

If no - Why do you think this is the case?

Reflective Motivation

Do you ever recall having thought to yourself that you may need some protection for your ears in relation to noisy activities, and then planned ahead?

If yes - How often would this happen?

Would it depend on the type of activity? Why?

If no - Any reasons why not?

Would it depend on the type of activity? Why?

Do you personally feel there are benefits to wearing earplugs?

If yes - Why and can you tell me what you think they are?

If no - Why do you think there are no benefits?

Do you personally feel there are restrictions to wearing earplugs?

If yes - Why and can you tell me what you think they are?

If no - Why do you think there are no restrictions?

Have any previous experiences made you decide that you want to wear earplugs?

If yes - Can you tell me about these experiences?

Did you act on it?

If no - On reflection, can you recall any experiences during or after a noisy activity that perhaps should have made you think about wearing earplugs?

Have you ever had the impulse during a noisy activity to suddenly want to wear earplugs?

If yes - Could you please elaborate as to why and where?

If no - Do you think there is any reason why not?

Have you ever had any personal positive experiences with earplugs?

If yes - Can you elaborate please?

If no - Any reason why this is?

Have you ever had any personal negative experiences with earplugs?

If yes - Can you elaborate please?

If no - Any reason why this is

Do you feel you can/could enjoy activities with earplugs?

If yes - Could you please elaborate as to why?

If no - Could you please elaborate as to why not?

[Ask at the end]

Do you plan to wear earplugs in the future?

If yes - Why is this?

If no - What are the reasons?

Has our conversation today made you think differently about wearing earplugs?

If yes - What particular points have made you think differently?

If no - Are there any reason why not?

Overall, do you have any positive or negative feelings towards earplugs and protecting your hearing in general? [Final thoughts on earplugs, positive or negative]

If yes - Can you elaborate please?

If no - Can you elaborate please?

Any final thoughts on earplugs and on getting people to wear them in the future?

That is the end of today's interview, thank you very much for taking part. Is there any further information that you would like me to discuss or help you with?
