# Supplementary material for: Identifying Targets for Interventions to Increase Earplug Use in Noisy Recreational Settings: A Qualitative Interview Study
Source: Int J Environ Res Public Health. 2021 Dec 7;18(24):12879. doi: 10.3390/ijerph182412879 (PMC8701360; doi:10.3390/ijerph182412879)
Supplement: Supplementary file 1 [file ijerph-18-12879-s001.zip › SF2 - Barriers and Enablers.pdf]

SF2– Barriers and Enablers

Barriers to recreational earplug use

Circumstantial (e.g., noise type or environment, volume, length of exposure, frequency)

Both ever- and never-performers referred to the fact that the circumstance attributes to their reasoning for not wearing earplugs, for example: expected loudness for certain physical environments; how often and how much time they are in that environment; and how loud they perceive it to be when in the environment. However, the activity may be loud enough to cause harm over time through cumulative noise exposure [23] (see '*lack of awareness and education*' and '*lack of prompts and cues*')**.**

> *"Maybe depends on the venue I guess, whether it's indoor or outdoor and how big the venue is and how close to…the audience are close to the speakers."* **P#17 (never)**

> *"No, I think I didn't take any action because with the nightclubs I still carried on. I didn't need earplugs. I think with the motorway, when I was driving on a motorway that was a routine habit already so it didn't change any of my actions in any way."* **P#3 (never)**

Detrimental impact on listening

Beliefs on the impact to listening and enjoyment differ between the two groups. Never-performers are speaking from a hypothetical point of view and almost all believe earplugs would diminish the overall listening experience, and communication.

> *"Yeah, like, the issue is that it's too loud, and putting plugs in or headphones on will make the whole thing quieter, but I might as well just not be listening."* **P#6 (never)**

While ever-performers had both factual and hypothetical experiences ranging across various activities, their views were generally not as negative (see '*perceived benefits of using earplugs*'). Factual experiences were mainly from recreational musicians, and it is well documented that

musicians struggle with the transition after years of playing without them [46]. However, those that had not yet tried at live music events had similar hypothetical preconceptions as never-performers.

> *"I would say concerts and theatres but I feel like it would detract too much from the experience of it."* **P#14 (ever)**

Ever-performers referenced foam earplugs as being lower quality compared to high fidelity earplugs. However, it did not prevent people from using them.

> *"And I know that the foam earplugs, I've used them once or twice before even that happened at gigs and so on, because they can take away some of the loud bass that prevents you from hearing the actual…the words to songs that are being played or whatever."* **P#18 (ever)**

Forgetting

Ever-performers discussed instances of forgetting earplugs. Forgetfulness can be seen as a resource issue as within many environments, it is not be possible to get another pair, and the individuals will continue to place themselves at risk, even though they are aware of the benefits of earplugs. Forgetfulness is also a consequence of lack of motivation (see '*not automatic to use earplugs*').

> *"I do have some earplugs that I can use, but I'm very bad at remembering them. Yeah, by the time I'm out of the door I've forgotten them, then I'm out and that's it. So, yeah, I would know the right way to use them if I could only remember to take them."* **P#5 (ever)**

Incorrect use

As expected with anyone who has not used earplugs, never-performers have an element of not fully acquiring the physical skill of correct placement, and evidence suggests that this can have

an impact on the level of protection provided [30]. However, many believe this not to be a major concern (see '*ability to use earplugs*').

> *"I never actually tried it myself."* **P#7 (never)**

Lack of awareness and education

Both groups indicate a certain lack of awareness. Never-performers are carrying out risky listening behaviours, but they are aware their behaviour is causing temporary hearing symptoms. However, they are lacking the awareness to protect themselves to stop it occurring. Other factors besides awareness may be involved, see '*lack of prompts and cues*' and '*poor accessibility*'.

> *"Yeah, and I have…like, I get sort of mild tinnitus as well so, you know, I've sort of looked up for stuff around that and gone, just how much have I messed up my hearing by my wild and crazy lifestyle? Is it going to go away, am I stuck with it?"* **P#9 (never)**

This lack of awareness links into the issue of knowing 'how loud is too loud' (see '*circumstantial*' barrier) and ever-performers responses suggest they struggled with this aspect.

> *"It's not as loud, I mean, it's not, yeah, it's not as loud, but it certainly is loud enough that someone would need…you'd need to certainly raise your voice or shout for, definitely, me to hear you"* **P#6 (never)**

No participants were able to refer to any form of formal education on hearing health and promotion of hearing protection (e.g., earplugs).

> *"I like to keep healthy, but I guess I haven't really seen my ears as part of my health as such in a sense, like I eat healthy and stuff like that, fruit and veg, but I haven't really considered…there's not much about – well, there's not much promotion about the dangers of noise"* **P#20 (never)**

Lack of comfort

Never-performers suggest earplugs would be uncomfortable. In the main, this is hypothetical, but there are those that have tried foam earplugs for sleeping, and found it uncomfortable or a strange sensation. These preconceptions are harmful to recreational earplug use, and therefore, first impressions are important. Alongside the belief that earplugs will alter the experience (see '*detrimental impact on listening*'), preconceptions about earplug use are compounded, which further prevents the uptake of earplugs. This is also a resources issue for never-performers, due to beliefs that the resources available are uncomfortable (see '*poor accessibility*').

> *"Well, bearing in mind I'd bought cheap ones from Amazon, but they weren't comfortable, and they fell out and they made it hard to sleep."* **P#6 (never)**

Lack of shared experiences

Never-performers did not discuss shared experiences of hearing symptoms, noise exposure, and earplugs. They have not had any social facilitators/credible sources in their lives (e.g., friends, family, peers) to observe or gain advice on these topics. This also links into '*lack of awareness and education*' as many never-performers have had personal experiences of symptoms in the past, but not acted on this.

> "*Yeah, I mean, like I said, I've never even thought about it for protection and I don't know what it'd be like. I've never had any friends say, hi, you should try this as well, but sometimes peers conversations is very helpful to gauge what things are like*." **P#8 (never)**

Lack of prompts and cues

No participant could recall ever seeing any physical prompts or cues while taking part in noisy recreational activities. In the UK it is recommended that if a live music event is thought to average an A-weighted continuous noise level over the course of the event (LAeq) of 96 dB then

patrons are to be made aware so they can take precautions [33]. Prompts and cues can take many forms, such as, warnings on noise levels, promoting earplugs, self-affirmation, and advice and encouragement, to name a few. However, the lack of not only presents barriers for opportunities, but also motivation (see '*not an automatic to use earplugs*').

> *"No, there's never any. There's always about strobe lights, if they use strobe lights, they always have a health warning, but they never have a health warning about hearing, not that I've seen."* **P#2 (ever)**

Low priority

Hearing health is a low priority (e.g., taken for granted) and has resulted in a lack of desire and impulse to protect it (see '*not automatic to use earplugs*'). Including use of earplugs, even though people know the benefits (see '*perceived benefits of using earplugs*').

> *"It's just something you take for granted, isn't it, your hearing? And you don't ever really think of it until it becomes a problem and you've got, like, ringing in your ears or something."* **P#15 (never)**

Never-performers state that they do not consider using earplugs before any noisy activity.

> *"No, I'd put my fingers in my ears but it still wouldn't have crossed my mind to get earplugs."* **P#12 (never)**

Not automatic to use earplugs

For all those not using earplugs automatic responses will need to be built upon using reinforcement tactics (rewards and incentives), and the use of emotions (positive/negative affect) to evoke a willingness to change behaviour.

> *"The closest to that impulse maybe would be me covering my ears, but after a few seconds of getting used to the noise, it'll be gone."* **P#7 (never)**

No preventative planning

Never-performers do not plan any protective behaviour, and they do not intend to change their behavioural plans yet. Indicating a lack of goals, intentions and beliefs about consequences.

> *"Probably, but not in the near future, maybe in 10-15 years' time when, I don't know, I'll be thinking, okay, I'm going to grow old soon, I might as well try to preserve my hearing."* **P#7 (never)**

Poor accessibility

Never-performers discussed that they would access earplugs by going to the chemist/pharmacy. Although earplugs are available within these stores resources are usually limited (see '*detrimental impact on listening*'). Accessing earplugs in this manner is also of no benefit if the person is already in attendance at an event. The current theme links into '*lacking awareness and education*'.

> *"I'd probably go to the chemist or the supermarket and hunt around the shelves."* **P#12 (never)**

Social stigma, norms, conformity etc

Both groups identified with social influences being barriers. There were those that felt earplugs were not socially accepted or "*might look weird*". Ever-performers stated they felt self-conscious, that passers-by made comments, and some wanted to hide the fact that they were wearing earplugs (e.g., aesthetics/noticeable). Never-performers said not observing others wearing earplugs makes it harder. These social influences also cause motivational barriers for both groups (e.g., social role and identity).

> *"I've been told off for it a couple of times."* **P#10 (ever)**
>
> *"I think some people probably think you might look weird if you do it, especially in the night club."* **P#19 (never)**

"Judgemental, yeah. So definitely when I was younger, I'd put them in before I got to somewhere or go to the toilets in the venue and put the earplugs and then wear my hair down so it would hide them." **P#21 (ever)**

Enablers to recreational earplug use

Ability to use earplugs (practical and theoretical)

Ever-performers are confident that they have the physical skills to perform the behaviour to a high standard, as they have encountered no problems to date. However, similar to the barrier '*incorrect use*' for never-performers, we have to assume ever-performers are doing this correctly, and achieving appropriate levels of protection [30].

"Yeah, [there was a little tool] to like…a little tool then it helps you get them into the right position, or where it feels comfortable. So, I think I got it all right." **P#1 (ever)**

Advice and encouragement (social facilitators/credible sources)

Ever-performers discussed receiving advice from credible sources/social facilitators about hearing symptoms and use of earplugs. Helping them to alter their behaviour.

"My younger brother, he's…or at least at the time he was a drummer in a metal band, or two or three metal bands probably at the time. So he'd kind of come along to the gig with me and my partner at the time and had gone, oh yeah I've got these earplugs that I use when I'm drumming and I just take them to all gigs now, and it's really great because you can still hear the band but when you come out of it you're not completely deaf. And I was like, oh that actually seems alright, you know, that could…that's probably the thing to do." **P#9 (never)**

Never-performers did not mention such influences (see '*lack of experiences*'). However, they did discuss how exposure to people they deem 'credible' might help them consider using earplugs.

> *"I suppose if I went to do a hearing test and I would be told, right, the way it looks like right now, it seems that you actually need to start wearing earplugs, otherwise your hearing can start going bad, really, really bad in the next 15 years, that would freak me out a little bit and I will say, okay, let's look into this."* **P#7 (never)**

The interview process made never-performers consider their own behaviour, perhaps indicating the value of credible sources. Talking and thinking objectively about a subject makes the individual reconsider, without the credible source having to re-educate (see '*awareness of NIHL and hearing protection behaviours*').

> *"This conversation has. Just because I don't really think about it, but it's kind of stupid not to, because it's a really easy thing to do, just put some earplugs in to prevent you damaging your ears, but it's just not something that you think of. Well, I really ever think about."* **P#15 (never)**

Both groups discussed the fact that seeing other people using earplugs may make them want to wear them (e.g., positive group norms). This could be family members, friends, children, or complete strangers.

> *"Yeah, maybe I would start using them if friends and family started."* **P#20 (never)**
>
> *"I think when I was at university I was going to a lot of gigs, I was going to maybe three or four a week, sometimes I'd go to two different gigs on a night. So, I think it was just from talking to other people or just noticing that other people were wearing earplugs when I started to think, oh maybe I should do that as well."* **P#21 (ever)**

Aesthetics not important

Never-performers felt that even though they have reservations about using earplugs, aesthetics (e.g., appearance) would not stop them from wearing.

"I think if I wanted to do something that was really noisy and I wanted to do it enough, like I really wanted to see the band or whatever and I knew it was going to be a very noisy and a very small environment and all the rest of it, I would put up with the look of it to be able to have the experience. It's not something that bothers me particularly." **P#4 (never)**

Affordable

Ever-performers stated that they find using earplugs an affordable behaviour.

"Yeah, I mean, I was expecting them to be more expensive. I mean, I know they're only little things but I mean, you can get really expensive ones if you really want. But yeah, I was pleasantly surprised that they weren't too expensive for decent-ish ones." **P#1 (ever)**

Availability

Both groups discussed the availability of earplugs within different contexts/environments would perhaps encourage use, and/or uptake. Ever-performers thought availability would help if they had forgotten their own earplugs, or to try in contexts that they previously have not, and never-performers would perhaps try for the first time.

"They all seem to have them behind the bar for bar staff, so yeah. I tend to go to…and will ask. I mean, even now, if I've forgotten the specific ones that I've got that I take to a gig, kind of thing, oh, if I've had them in a different bag to take to roller derby stuff and I've forgotten to take them out with me, then I will go and get the foam ones as they…well, it's better than nothing" **P#18 (ever)**

"If they were offered at the entrance, I would probably think, okay, maybe that's particularly loud and maybe I should wear these" **P#7 (never)**

Awareness of NIHL and hearing protection behaviours

Both ever- and never-performers appear to have adequate knowledge and awareness that noise causes hearing loss, that certain situations are too loud, and that hearing protection could help.

> *"I mean, you do need to protect your hearing. Because obviously, I mean, prolonged exposure to loud noise is going to damage your ears."* **P#1 (ever)**
>
> "*My understanding of it is that if you do it consistently, especially when you're young, so around my age, it will sort of delay hearing loss when you're growing older. It's obviously not the only factor, but it's one of the factors that will sort of make it less likely for you to become impaired or in terms of hearing when you become older, if you take measures early to protect your hearing, that's my perception of it*." **P#1 (ever)**

Ever-performers are given a certain amount of 'peace of mind' through performing the behaviour, indicating a more than adequate level of knowledge and awareness of noise induced hearing loss and the dangers of noise.

> *"I know this is going to be an unpleasant sensory experience if I don't, so I will. So, I guess, it's peace of mind but more…less in the sense of preserving my hearing for some older me down the road, and more in the sense of preserving it for myself in the moment."* **P#13 (ever)**

However, ever- and never-performers still require more specific awareness information, such as, 'how loud is too loud' within certain environments (see '*lack of awareness and education' and barrier 'circumstantial'*).

Circumstantial (e.g., noise type or environment, volume, length of exposure, frequency)

Similarly, to barriers (see '*circumstantial*' barrier) the circumstance that both groups find themselves in can also act as an enabler. The environmental changes in this instance can be

interpreted that earplugs would be beneficial to block out unwanted noise, for example, motorsport noise; on the other hand, it could be deemed useful for changes in certain types of musical noise over another. The range of answers indicates the variety of beliefs that different people deem a 'loud' and potentially appropriate environment to use earplugs. Never-performers are speaking hypothetically and ever-performers from experience.

> *"Depends on kind of what we are attending, the meaning of earplugs might be different. For example how sound is attractive to that activity I think. For the music, the sound is essential. But yeah, for the other events, for example motor sports or even watching sports events, the vision itself is more important than noise, maybe."* **P#17 (never)**
>
> *"It depends on the gig, there are some gigs where going in I know I'm going to want them throughout, so I'll put them in. Others I may, sort of you know… say there are three or four bands playing, I may know which ones I'll need it for and which ones I don't, I'll make that decision when they start."* **P#13 (ever)**

Comfort

Ever-performers referred to finding earplugs comfortable. The physical feeling of comfort by ever-performers counteracts the '*lack of comfort*' concern suggested by never-performers.

> "When they're actually in my ear they felt fine. Comfortable enough. I mean, I've got musician earplugs so I'd like to hope they were designed to be comfortable." **P#1 (ever)**

Earplug performance

Ever-performers mentioned that they still enjoyed activities while performing the behaviour, which is in contrast to the perceived barrier '*detrimental impact on listening*'.

> *"When I've worn them, I can, definitely…you can hear the benefit because you…like I mean, they seem pretty decent, you can still hear everything it doesn't…you don't lose any of the music. You can still hear and talk to people"* **P#5 (ever)**

"I can enjoy what I'm trying to hear better without being overwhelmed by a wall of sound" **P#13 (ever)**

Earplug routine and planning

Ever-performers discussed routines in some context. There were those that had a routine in place to help them remember their earplugs, including physical carrycases, while others recognised that this is perhaps something they need to adopt. Creating plans and habits leads to permanent changes in behaviours (see '*no preventative planning*' and '*not automatic to use earplugs*').

"In terms of actually taking them from my house to a game or a practice or whatever, then I have a bum bag or fanny pack. So, I have one specifically for roller derby. So, I just keep them in there when I'm not using. They come in a little plastic case. So, I just keep them in that, in the plastic case the whole time. if I remember to take them before I go out to a gig then I will get them out of the bum bag and then hang that on the door, so when I get back in again, then the bag is there and really obviously reminding me to put them back in again afterwards." **P#18 (ever)**

Experiences with hearing symptoms

Ever-performers have had previous experiences of hearing symptoms that has aided earplug use, either their own, or advice from what they would deem credible sources (see 'advice and encouragement'). Whereas never-performers who have had symptoms have not acted, (see '*lack of awareness and education*'). This not only highlights knowledge and decision processes (see '*awareness of NIHL and hearing protection behaviours*'), but also beliefs about consequences and optimism that earplugs will help.

"*So we went to a concert and I couldn't hear, I was playing Sunday league football, and I went to the concert on the Saturday night, and I couldn't hear anything, 'because the music was obviously so loud. And that's when my mum said, well you should be really*

thinking about protecting that. She wears hearing aids, so she's all for it, protecting hearing." **P#2 (ever)**

Good quality earplugs

Ever-performers discussed that the better the quality earplugs, then the better the experience. This ranged from people saying they preferred mid-range (e.g., musician style/high fidelity) earplugs, and that foam earplugs are bad for the environment. However, it must be noted that there were people who found the use of foam earplugs to be acceptable, but they had the knowledge that they could be improved upon (see '*knowledge about earplugs*').

> *"So I think it does make a difference and, you know, the results could be better so I would say it's advisable that you buy some good quality earplugs because it could place a benefit."* **P#3 (ever)**
>
> *"Yeah, it's made me think I'll probably buy some more permanent ones instead of just relying on the foam disposable ones. That's also because I care about the environment and I don't want to keep using ones that you just throw away; it seems a bit wasteful."* **P#21 (ever)**

Knowledge of earplugs

Both groups discussed knowledge on specific details of earplugs, including, range of prices, different designs (custom, flanged, foam), and where to get them. However, ever-performers' knowledge appears more in depth (see '*poor accessibility*'), as they knew more about the range (musician/custom), prices, and searching the internet to find the best quality. Never-performers were more restricted to theoretical knowledge of foam earplugs (see '*awareness of NIHL and hearing protection behaviours*', '*earplug performance*' and '*good quality earplugs*').

> *"But there were quite a few different ones to pick from. And so, I was like, right, I'll just get one…I just Googled good musician earplugs, so I got one of those ones that was made and recommended, so…I think it was between 15 and 20 quid."* **P#1 (ever)**

Although, never-performers have never used earplugs recreationally, they believe they have the knowledge and skills to perform the behaviour correctly, and if they were unsure, they would read any appropriate information.

> *"…my guess is that if I google it I will find the answer."* **P#7**

Love of music

Those who wear earplugs for the context of music discussed that they want to be able to enjoy music for years to come, and that they cannot do without. This highlights stability of intentions and anticipated regret.

> *"I know that it's very important, because you don't want to lose your hearing, especially if you like music as much as me."* **P#2 (ever)**

Perceived benefits of using earplugs

Ever-performers found that they could still enjoy recreational activities with earplugs in place, highlighting that performance of the behaviour can relieve the anxiety or belief that earplugs will diminish enjoyment, which is a significant barrier for never-performers (see '*detrimental impact on listening*'). Those that are worried about damaging their hearing believe that using earplugs gives them peace of mind while still enjoying the activities they love (see '*awareness of NIHL and hearing protection behaviours*').

> *"When I've worn them, I can, definitely…you can hear the benefit because you…like I mean, they seem pretty decent, you can still hear everything it doesn't…you don't lose any of the music. You can still hear and talk to people"* **P#5 (ever)**

Prompts and cues

Ever-performers mentioned the potential benefits of prompts and cues. Their use would be pivotal in helping to remind them to wear their earplugs (see '*not automatic to use earplugs*'), or to

suggest that an activity is going to be loud enough to warrant their use (see '*lack of prompts and cues*'), and to perhaps inform them that earplugs are available (see '*availability*' and '*forgetting*').

> *"Maybe if they had the signs up, saying, this is a loud environment, protect your hearing, then, you know? That sort of reinforcement cue would help a lot."* **P#2 (ever)**
>
> *"And, so, I guess, if there's promotional material and little, you know, little footers on flyers for gigs and club nights and things about looking after your hearing."* **P#5 (ever)**
