# Supplementary material for: Identifying Targets for Interventions to Increase Earplug Use in Noisy Recreational Settings: A Qualitative Interview Study
Source: Int J Environ Res Public Health. 2021 Dec 7;18(24):12879. doi: 10.3390/ijerph182412879 (PMC8701360; doi:10.3390/ijerph182412879)
Supplement: Supplementary file 1 [file ijerph-18-12879-s001.zip › SF3 - TDF and COM descriptions.pdf]

SF3-TDF and COM descriptions

Clustered key TDF domain descriptions, and associated COM-B component descriptions; adapted from original sources [18, 20, 31].

**TDF domains**	**Description**
Social influences	Interpersonal processes that can cause individuals to change their thoughts, feelings, or behaviours towards using earplugs in noisy recreational contexts
Environmental context and resources	Any circumstance related to a person's situation, or environment that encourages or discourages the use of earplugs in noisy recreational contexts
Beliefs about consequences	Acceptance of the truth, reality or validity about an ability, talent, or facility of the benefits from the use of earplugs in noisy recreational settings
Memory, attention, and decision processes	The ability to retain information, focus selectively on aspects of the environment and choose between using or not using earplugs in noisy recreational contexts
Reinforcement	Increasing the probability of using earplugs in noisy recreational contexts by arranging a dependent relationship, or contingency, between the response and a given stimulus
Emotion	A complex reaction pattern, involving experiential, behavioural, and physiological elements, by which the individual attempts to deal with a personally significant matter or event
**COM-B components**	**Description**
Social opportunity	Opportunity afforded by interpersonal influences, social cues and cultural norms that influence the way that we think about using earplugs in noisy recreational contexts
Physical opportunity	Opportunity afforded by the environment involving time, resources, locations, cues, physical 'affordance' to use earplugs in noisy recreational contexts
Reflective motivation	Reflective processes involving plans (self-conscious intentions) and evaluations (beliefs about what is good and bad) to use earplugs in noisy recreational contexts
Psychological capability	Knowledge or psychological skills, strength or stamina to engage in the necessary mental processes to use earplugs in noisy recreational contexts
Automatic motivation	Automatic processes involving emotional reactions, desires (wants and needs), impulses, inhibitions, drive states and reflex responses to use earplugs in noisy recreational contexts
